# Fetal Vegf Genotype is More Important for Abortion Risk than Mother Genotype

**Published:** 2014

**Authors:** Sinem Atik Yalcintepe, Fatma Silan, Servet Ozden Hacivelioglu, Ahmet Uludag, Emine Cosar, Ozturk Ozdemir

**Affiliations:** 1 *Department of Medical Genetics, School of Medicine, Canakkale On Sekiz Mart University, Canakkale, Turkey.*; 2 *Department of Gynecology and Obstetrics, School of Medicine, Canakkale On Sekiz Mart University, Canakkale, Turkey.*

**Keywords:** Spontaneous abortion, VEGF, fetus, single nucleotide polymorphism

## Abstract

VEGF gene has been reported to be related with many diseases and recurrent pregnancy loss in various studies. Concerning the role of VEGF polymorphisms in pregnancy losses, generally mothers genotypes have been analyzed. To evaluate the association between VEGF A +405G/C (rs2010963), −460T/C (rs833061), +936C/T (rs3025039) and - 2578A/C (rs699947) polymorphisms and spontaneous abortion, we studied the genotypes of spontaneously aborted fetuses, their mothers and healthy controls. 23 spontaneously aborted fetal materials, 22 mothers who had these abortions and 86 healthy controls were included in this study. rs2010963, rs833061, rs3025039 and rs699947 polymorphisms were analyzed by Real Time PCR technique after genomic DNA isolation from all subjects. The frequencies of VEGF A rs2010963 GG genotype and rs2010963 G allele were higher in fetuses compared both with mothers and healthy controls. VEGF A rs3025039 TT genotype and rs3025039 T allele frequencies were higher in fetuses comparing with mothers. VEGF A rs833061 CT and TT genotypes frequencies were higher in fetuses comparing with mothers. We ascertained that VEGF A rs2010963, rs833061 and rs3025039 are the risk factors for spontaneous abortion in fetal genotypes comparing with their mothers and healthy controls.

Spontaneous abortion is defined as the loss of a fetus prior to 20 weeks of gestation. Approximately 15~20% of clinically recognized pregnancies are spontaneously aborted, most of them during the first trimester (1). The major cause of spontaneous abortion is fetal chromosomal abnormalies, contributing to about 50~60% of the cases among which chromosomal aneuploidy is the most prevalent (2). Other etiologic factors of spontaneous abortion are anatomic anomalies, endocrine or hormonal problems, coagulation protein defects, nutritional and environmental factors. Maternal serum b-hCG levels and sonographic parameters such as fetal heart rate and crown-rump length are used for prognosis and diagnosis of spontaneous abortion.

Vascular endothelial growth factor (VEGF) induces endothelial cell proliferation and migration, enhances vascular permeability, reduces endothelial cell apoptosis and promotes stromal proteolysis (3). The VEGF family includes placenta growth factor, VEGF-A, VEGF-B, VEGF-C, VEGF-D, and VEGF-E. VEGF A is the most potent angiogenic factor.

The VEGF-A gene is located on chromosome 6p21.3 and contains eight exons and seven introns. Several single nucleotide polymorphisms (SNPs) have been identified in the VEGF-A gene and are believed to have functional activity. Among these, −460T/ C (rs833061) and -2578A/C (rs699947) in the promoter region, +405G/C (rs2010963) in the 5′-untranslated region (position+ 405 after transcription initiation site), and+ 936C/T (rs3025039) in the 3′- untranslated region are known to modulate the protein expression of VEGF-A (4-6).

VEGF plays an important role in fetal and placental angiogenic development. VEGF also plays a major role in oocyte maturation, embryo implantation/ development, decidualized endometrial vascularization and placenta angiogenesis/ vascularization in early gestation (7). In early pregnancy, the status of chorionic villi vascularization is related to embryonic development, and reduced placental trophoblastic VEGF level has been described in the decidual endothelium of spontaneous miscarriages (8). Several VEGF polymorphisms have been reported to affect VEGF activity and expression (4, 9).

Various studies have investigated the association of VEGF gene polymorphisms with diseases in which angiogenesis plays a major role in pathogenesis, such as diabetic retinopathy (4), renal cell carcinoma (10), acute renal allograft rejection (6), malignant melanoma (11) and other malignancies (12, 13). The results, however, were mixed. It is reported that VEGF A -2578 AA genotype is a risk factor and -2578 CC genotype is a protective factor for myocardial infarction (14). VEGF -460C/T polymorphism was reported as a severe risk factor for retinopathy (15) and +405 GG genotype as a risk factor in primary glomerulonephritis (5). A relationship between *VEGF* gene and breast cancer was reported in different studies. Accordingly, the TT genotype for +936C/ T polymorphism reduces breast cancer risk (12). In different studies, a relationship between VEGF -1154G/ A, -2578C/ A, +936C/ T polymorphisms and recurrent pregnancy loss were reported (3, 16). Abortion etiology is still mostly unknown, but genetic factors are the most effective group. We hypothesized that VEGF A polymorphisms in fetal genotype could be risk factors for spontaneous abortion and analyzed +405G/C, −460T/ C, +936C/ T and -2578A/ C polymorphisms in this regard.

## Materials and Methods

**Patients and clinical diagnosis**

23 abortion materials and their 22 mothers (one of the mothers had two abortions), and control group including 86 fertile healthy controls (43 couples with no abortion history) were included in this study. Abortion materials were obtained from only spontaneous abortions. Control group subjects had at least two children and no spontaneous abortion. Maternally contaminated abortion materials were excluded from the study (50 abortion materials were taken for this study). Maternal contamination was detected with the correlation of fetus and mother STR markers. 131 cases including 23 abortion materials, 22 mothers and 86 controls were genotyped for VEGF A SNPs (+405 C/G, -460 C/T, +936 C/T and -2578 C/A) and both abortion materials group and the corresponding mothers group as well as abortion materials group and healthy controls were compared.

**Mutation analysis**

Total genomic DNA was isolated from 10-15 mg of abortion material tissue by the QIAGEN QIAamp DNA Mini Kit and peripheral blood samples containing EDTA from the mothers and the control group by the QIAGEN QIAamp DNA Blood Mini Kit. Four polymorphic regions of the VEGF A gene (+405 C/G, -460 C/T, +936 C/T and -2578 C/A) DNA fragments were ampliﬁed by real-time polymerase chain reaction technique using LightCycler 2.0 (Roche ).

**Fig 1:**
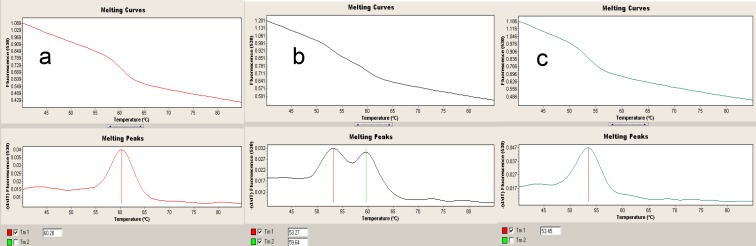
VEGF A -460 C/T polymorphism CC genotype (a), CT genotype (b) and TT genotype (c) profiles in Real Time PCR

To determine the VEGF +405 G/C, -2578 C/A, +460 C/T, and +936 C/T polymorphisms, genomic DNA was amplified using the primers (F5'-ATTTATTTTTGCTTGCCATT-3') (R5'-GTC-TGTCTGTCTGTCCGTCA-3') (F5'-GGATGGGG-CTGACTAGGTAAGC-3') (R5'-AGCCCCCTTTT-CCTCCAAC-3'), (F5'-TGTGCGTGTGGGGTTG-AGCG-3').(R5'- TACGTGCGGACAGGGCCTGA-3'), and (F5'-AAGGAAGAGGAGA-CTCTGCGC-3') (R5'-TATGTGGGTGGGTGTGT-CTACAG-3'), respectively. Brieﬂy, LightCycler FastStart DNA Master HybProbes , master mix  (water, PCR-grade, Mg^+2^, stock solution, Primer mix, and HybProbe mix), and DNA template ()  were used for real- time ampliﬁcation. The multiple PCR consisted of a denaturation step of 10 min at 95^0^C, followed by 45 cycles of 10 s at 95^0^C,10 s at 60^0^C, and 15 s at 72^0^C, and a melting step of 20 s at 95^0^C, 20 s at 40^0^C, a continuous mode at 85^0^C, a cooling step of 30 s at 40^0^C for all four regions. A software program (LightCycler 2.0, Roche) was used for the detection of the genotype proﬁles of the target gene. VEGF A +405 C/G (rs2010963) wild type +405 CC was analyzed in channel 530 and with Tm 65 ^0^C. The mutant genotype +405 GG was analyzed with Tm 52 ^0^C. VEGF A -460 C/T (rs833061) wild type -460 CC was analyzed in channel 530 with Tm 61 ^0^C. The mutant genotype* -*460 TT was analyzed with Tm 54 ^0^C (Figure 1).

VEGF A +936 C/T (rs3025039) wild type +936 CC was analyzed in channel 530 with Tm 66 ^0^C. The mutant genotype +936 TT was analyzed with Tm 57 ^0^C. VEGF A -2578 C/A (rs699947) wild type -2578 CC was analyzed in channel 530 with Tm 55 ^0^C. The mutant genotype -2578 AA was analyzed with Tm 62 ^0^C.

**Statistical analysis**

Statistical analysis was done using medcalc statistical program. Chi-square test was used to analyze the differences between the abortion materials, mothers and control subjects. Genotype and allele frequencies were compared between fetal (abortion materials) group and mother group, and fetal group and fertile control group. Odds ratio (OR) and p-values were used to estimate the risk for the VEGF A SNPs in the groups.

## Results

By real time PCR technique, we evaluated four SNPs of the *VEGF A* gene in study groups (23 abortion materials, 22 mothers and 86 control subjects), and the results were compared between both fetus-mother group and fetus-healthy control group. The estimate risk was examined by odds ratio.

Peripheral blood-EDTA samples from mothers and healthy controls and abortion material tissue samples were examined for genotyping in the current study. The mean age of the mothers was 29.6 (21-40) years, and the mean age of controls was 46.5 (27-68) years. The mean abortion week was 9.4. Twenty abortions were in the first trimester and three abortions in the second trimester. There were no other diseases in the study groups.

The genotype analysis and statistical results for VEGF A +405 C/G, C+936 C/T, -460 C/T and -2578 C/A are presented in table 1. In all statistical analyses, we performed four different comparisons: 1. Percentages of wild genotype and heterozygous+ homozygous genotypes. 2. Percentages of wild genotype+ heterozygous genotype and homozygous genotype. 3. Percentages of wild genotype and homozygous genotype. 4. Allele frequencies. For *VEGF A* +405 C/G polymorphism, in the statistical analysis GG genotype and G allele were found as risk factors for abortion in both groups (Table 1). GG genotype represents 2.5- 4.3 fold risk in fetus group comparing with mothers and control group.

For *VEGF A* +936 C/T polymorphism, in the statistical analysis TT genotype and T allele were found as risk factors for abortion in fetus compared to their mothers and controls (Table 1). TT genotype had 4 fold risk in fetus group comparing with mothers. The difference in genotypes frequencies between fetus and control group was not statistically significant (Table 1).

For *VEGF A* -2578C/A polymorphism, according to the statistical analyses none of the genotypes and alleles were risk factors for spontaneous abortion (Table 1).

For *VEGF A* -460 C/T polymorphism, the sum of CT+ TT genotypes was found as a risk factor for abortion while comparing fetuses with their mothers with an odd ratio of 2.3 (Table 1). The difference in genotypes frequencies between fetus and control group was not statistically significant (p>0.05) (Table 1).

## Discussion

VEGF has important roles in vascular permeability and angiogenesis, and regulates multiple endothelial cell functions. VEGF induces endothelial cell proliferation, migration and differentiation, and stimulates endothelial cell survival. Depending on its angiogenic and mitogenic properties, VEGF is important in embryonic development. The human placenta is rich in angiogenic factors such as VEGF, which plays an important role not only in forming placental vessels, but also in maternal vascular adaptation to pregnancy. In the placenta, vasculogenesis, the de novo synthesis of new blood vessels, accounts for the majority of new vessel formation during the first trimester, and is initially observed around day 21 post-coitum (17). In our study, the abortions occured mostly in the first trimester (20 (87%) of 23 abortions) as an evidence of this knowledge. A role for VEGF in embryonic development and trophoblast vascularization was determined (18), and altered expression of VEGF and its receptors were reported in placental and decidual samples from early abortions (8).

Functional polymorphism of VEGF may also influence prematurity by the alteration of VEGF production. Watson et al. demonstrated that in the presence of the VEGF +405 CC genotype, the peripheral blood mononuclear cells produce less VEGF than those with the GG genotype (19). VEGF gene has not only an effect on abortions, but it also seems to be related to many pregnancy complications (20, 21).

**Table 1:** VEGF A polymorphisms (+405 C/G, -460 C/T, +936 C/T, -2578 C/A) genotype numbers, percentages, and statistical analyses betweem fetal materials, mothers and control subjects genotypes

**Polymorphisms of ** ***VEGF A***	**Groups**									**Significance**			
	**Fetal Material (n/%)**			**Mothers (n/%)**			**Controls (n/%)**			**Compared groups**	**P value**	**OR**	**95 % CI**
**+405C/G**	CC	CG	GG	CC	CG	GG	CC	CG	GG	FvM and FvC			
	1/4.3	10/43.5	12/52.2	2/10	12/60	6/30	9/10.5	51/59.3	26/30.2	CC+CG and GG	0.0017	2.5278	1.4148-4.5164
**AF of C and G**	C:0.26		G:0.74	C:0.40		G:0.60	C:0.40	G:0.60		CC and GG	0.0208	4.3333	1.2495-15.0280
										AF C and G	0.0363	1.8974	1.0416-3.4566
**-460C/T**	CC	CT	TT	CC	CT	TT	CC	CT	TT	FvM			
	2/10	12/60	6/30	5/21.7	11/47.8	7/30.5	18/20.9	48/55.8	20/23.3	CC and CT+TT	0.0351	2.3924	1.0628-5.3856
**AF of C and T**	C:0.40		T:0.60	C:0.46		T:0.54	C:0.49	T:0.51					
**+936C/T**	CC	CT	TT	CC	CT	TT	CC	CT	TT	FvM			
	12/60	5/25	3/15	16/69.6	6/26.1	1/4.3	66/76.7	19/22.1	1/1.2	CC+CT and TT	0.0131	4.2353	1.3533-13.2551
**AF of C and T**	C:0.725		T:0.275	C:0.830		T:0.170	C:0.880	T:0.120		CC and TT	0.0123	4.3750	1.3775-13.8947
										AF C and T	<0.0001	1.8519	1.4927-2.2976
**-2578C/A**	CC	CA	AA	CC	CA	AA	CC	CA	AA	No significance			
	7/30.5	13/56.5	3/13	6/30	12/60	2/10	20/23.3	48/55.8	18/20.9				
**AF of C and A**	C:0.59		A:0.41	C:0.60	A:0.40		C:0.51	A:0.49					

*OR: Odds ratio, AF: Allele Frequency, FvM: Fetus versus Mother, FvC: Fetus versus Controls Statistics: medcalc statistic programme*

In this report, we studied *VEGF A* +405 C/G, +936 C/T, -460 C/T polymorphisms in relation with spontaneous abortion risk. *VEGF A* +405 GG genotype and the G allele are risk factors in fetal genome according to comparisons made with their mothers and healthy control group genotypes. *VEGF A* +936 TT genotype and the T allele are risk factors in fetuses when compared with their mothers. *VEGF A* -460 CT and -460 TT genotypes in fetuses are together increasing the risk of abortion as compared with their mothers. *VEGF A* -2578 C/A polymorphism was not found to be a risk factor for spontaneous abortion in this study.

*VEGF* gene studies about abortions, especially consider recurrent pregnancy losses. Samli et al. studied *VEGF* -1154 G/A, -2578 C/A, -460 C/T and +936 C/T polymorphisms in 38 recurrent pregnancy loss (RPL) patients and 30 controls. They reported a relationship between -1154 G/A polymorphism and RPL and proposed that miscarriages would be due to VEGFs' importance in placental angiogenesis (22). Su et al. also reported a relationship between *VEGF* -1154 G/A polymorphism and RPL (16). Traina et al. reported that there is no relationship between *VEGF* -634 G/C and +936 C/T polymorphisms and RPL (23). Lee et al. reported a relationship between RPL and -2578 C/A, -1154 G/A, -634 G/C, +936 C/T polymorphisms with 215 RPL patients and 113 controls (24). Although various *VEGF* gene and RPL studies are found in the literature, but these studies were planned with mothers genotypes. In our study, we correlated fetus, mother and control subjects genotypes for spontaneous abortions. A haplotype study which compared the -2578 CA+ AA/ -634 CC and -1154 GA+ AA/ -634 CC combined genotypes of spontaneous abortion materials with control subjects reported these genotypes in the fetus as possible risk factors for spontaneous abortion (25). Thus, our study is the first one reporting *VEGF A* +405 C/G +936 C/T, -460 C/T. Further studies and validations are necessary to determine the potential clinical impact of these findings.

Authors declare no conflict of interest.
